# Age Differences in the Relationship between Secondhand Smoke Exposure and Risk of Metabolic Syndrome: A Meta-Analysis

**DOI:** 10.3390/ijerph16081409

**Published:** 2019-04-19

**Authors:** Hui-Jian Chen, Gai-Ling Li, Ao Sun, Dang-Sheng Peng, Wan-Xia Zhang, You-E Yan

**Affiliations:** Department of Pharmacology, Wuhan University School of Basic Medical Sciences, Wuhan 430071, China; 2016203010030@whu.edu.cn (H.-J.C.); 2016203010033@whu.edu.cn (G.-L.L.); sunaonihao@foxmail.com (A.S.); 2018203010028@whu.edu.cn (D.-S.P.); zhangwanxia92@163.com (W.-X.Z.)

**Keywords:** secondhand smoke, metabolic syndrome, age difference, glucose metabolism, lipid metabolism, meta-analysis

## Abstract

Secondhand smoke (SHS), a common environmental exposure factor, has become a serious public health problem. Metabolic syndrome is another worldwide clinical challenge. Our study tried to determine the age differences in the relationship between SHS and the risk of metabolic syndrome. Studies were searched in PubMed and Web of Science from 11 November to 30 November 2018. Eighteen studies were finally included based on inclusion and exclusion criteria. The relationship between SHS and the risk indicators of metabolic syndrome was analyzed. The weighted mean difference (WMD) of fasting plasma glucose (FPG), insulin, body mass index (BMI), and waist circumference (WC), and the standard mean difference (SMD) of total cholesterol, triglycerides, and low- and high-density lipoprotein-cholesterol (LDL-C, HDL-C) were calculated in a meta-analysis. SHS was positively associated with the level of insulin and WC. According to the subgroup analysis based on age difference, SHS was positively associated with FPG in the upper age group, and positively associated with LDL-C and negatively associated with HDL-C in the lower age group. BMI showed a more obvious positive correlation in the adults group than in the children and the teenagers group. In conclusion, the association of metabolic syndrome with SHS varies with age. When exposed to SHS, older people may be more susceptible to glucose metabolic disorder, but younger people may be more susceptible to lipid metabolic disorder.

## 1. Introduction

Secondhand smoke (SHS), a common public health problem, refers to the burning tobacco products or gas exhaled by smokers. Globally, about 40% of children, 33% of adult male non-smokers, and 35% of adult female non-smokers were exposed to SHS in 2004 [1]. Smoking by household members or colleagues may be a major pathway for SHS. This kind of exposure is passive and easily overlooked. However, SHS caused around 600,000 deaths in 2004, among which 47% were women, 26% were men, and 28% were children [1]. In fact, SHS is associated with various diseases, such as heart disease [2], asthma [3], lung cancer [4], and cardiovascular diseases [5]. Recently, the effects of SHS on endocrine and metabolism diseases have been studied. According to Kim et al.’s study, exposure to SHS may be a risk factor for diabetes management [6]. Oba et al. reported that SHS decreases the sensitivity of insulin and pancreatic β-cell function and appears to be associated with diabetic states [7].

Metabolic syndrome is one of the major public health and clinical challenges worldwide. In the United States, 4.5% of adolescents, 35% of adults, and as much as 50% of the people aged over 60 years suffer from metabolic syndrome [8,9]. Metabolic syndrome, according to the National Cholesterol Education Program (NCEP) criteria, is a group of three or more risk factors, which include abdominal obesity, dyslipidemia such as low levels of high-density lipoprotein cholesterol (HDL-C) and high levels of triglycerides, high blood pressure, and elevated fasting plasma glucose (FPG) [10,11]. Many factors may induce metabolic syndrome, including unhealthy eating habits, short sleep duration, and lack of exercise [12,13,14,15], but the effects of SHS on metabolic syndrome have not been fully studied. In our meta-analysis, we analyze the relationship of SHS with some risk indicators of metabolic syndrome in young people and adults, such as fasting plasma glucose (FPG), insulin, total cholesterol, triglycerides, low-density lipoprotein cholesterol (LDL-C), HDL-C, body mass index (BMI), and waist circumference (WC). Moreover, a subgroup analysis based on age difference is conducted. SHS, as a common environmental factor, may be a potential factor that induces metabolic syndrome, but the harm caused by SHS is easily overlooked. Our study focuses on these two important public health issues and tries to explore the links between SHS and metabolic syndrome and potential differences between age groups. In doing so, this contributes to gaining a deeper understanding of the passive effects of SHS. Currently, no relevant review has been published.

## 2. Materials and Methods

### 2.1. Search Strategy

Articles were identified by searches of PubMed and Web of Science, and all documents were collected from 11 November to 30 November 2018.

The following key words were used:

#1 Environmental tobacco smoking OR passive smoking OR secondhand smoking OR household smoking;

#2 Free fatty acids OR total cholesterol OR triglycerides OR HDL-C OR LDL-C OR glucose OR insulin OR blood biochemistry OR body weight OR BMI OR waist circumference OR obesity OR overweight;

#1 AND #2.

### 2.2. Study Selection

Studies were imported into EndNote X7 (Thompson Reuters, San Francisco, CA, USA), and duplicate records were removed. All articles were screened independently through titles and abstracts by two reviewers (H.J. Chen and G.L. Li), and relevant full-texts were obtained.

Data were extracted from the appropriate studies that met the inclusion criteria listed as follows: (1) human studies; (2) the methods and objectives of each study are similar; (3) the results of the survey include at least one set of data; (4) the measurement data provides the means, standard deviation, and sample size; and (5) the study type is a cohort study or cross-sectional study.

The exclusion criteria included (1) repeating studies; (2) studies are defined as low quality in a quality assessment; (3) absence of some key information; (4) the data of the investigation and study are not completed through a document delivery service and full text cannot be obtained by emailing the authors; (5) the statistical method used is incorrect and cannot be modified, and the measurement data do not provide means ± standard deviation and sample size; and (6) studies were not based on humans.

### 2.3. Data Extraction

The main extracted information included the name of the first author, the publication year, the type of study, the sample size, the study period; the methods of SHS assessment; the average age of participants; the levels of FPG, insulin, total cholesterol, triglycerides, LDL-C, and HDL-C; BMI, and WC. The data had to be converted into the same units if the units were not consistent between studies. For example, the units for WC could be transformed from cm into mm. Some articles with more than one group of age-specific data were included in the subgroup analysis of age difference.

### 2.4. Quality Assessment

The quality of each study was independently evaluated by two authors (H.J. Chen and G.L. Li), and discrepancies were resolved by discussing with the third author (A. Sun). The quality of each cross-sectional study was evaluated by the Agency for Healthcare Research and Quality (AHRQ): low quality = 0–3; moderate quality = 4–7; high quality = 8–11 [16]. The quality of each cohort study was assessed by the Newcastle-Ottawa Scale (NOS) assessment, and scores ranged from 0 (lowest) to 9 (highest), and a study earning a score of seven or more was considered to be of high quality [17].

### 2.5. Statistical Analysis

Stata12.0 software (Stata Corporation, College Station, TX, USA) was used for meta-analysis. Continuous variables were presented as the weighted mean difference (WMD) with 95% confidence interval (CI). If the data could not be converted into the same units, the variables were switched to the standard mean difference (SMD) with 95% CI. When the diamond fell to the left or right of the invalid vertical line, it was considered indicative of statistical significance. No statistical difference referred to the diamond crossing with invalid vertical line.

The Q statistic (*p* < 0.10 indicating significant heterogeneity) and the I^2^ statistic (I^2^ > 75.0%, 50.0–75.0%, and < 50.0% indicating substantial, moderate, and low heterogeneity, respectively) were used to evaluate statistical heterogeneity [18].

Meta-analyses were conducted with fixed-effect models if no significant heterogeneity was detected by the Q statistic (*p* > 0.10, I^2^ < 50.0%), whereas random-effect models were applied under significant heterogeneity (*p* < 0.10, I^2^ > 50.0%). In addition, subgroup analyses were conducted to evaluate the effects of age. One-way ANOVA was used to evaluate the differences in BMI between subgroups, and *p* < 0.05 indicated significant statistical difference.

## 3. Results

### 3.1. Literature Search

**Figure 1:**
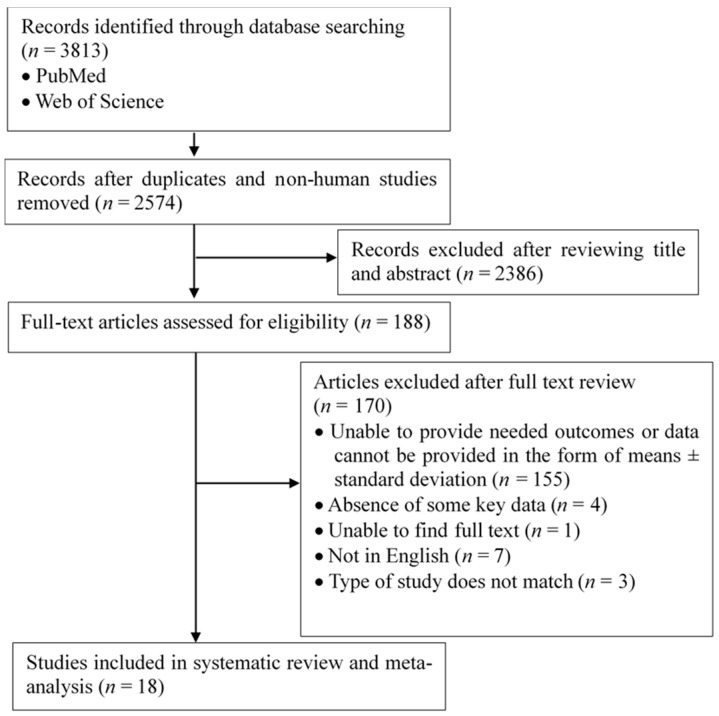
Flow chart of data sourcing and selection.

The initial literature retrieval identified 3813 papers from the PubMed and Web of Science databases. After initial screening based on titles and abstracts, 2386 papers that did not meet the inclusion criteria were excluded (non-human studies; reviews; studies not involving SHS; results were discontinuous variables; etc.), and 188 papers were obtained. Finally, a total of 18 studies were included in this study after carefully reading the full texts (Figure 1).

### 3.2. Study Characteristics and Quality Assessment

In this study, 18 studies were finally included, and their basic characteristics are shown in Table 1. Of the included studies, six of them were cohort studies, and the remaining 12 were cross-sectional studies. The ages of the individuals involved in these studies ranged from 1 to 74 years. Thirteen studies were of high quality, and five were of moderate quality (Table 2).

### 3.3. SHS and Disorder of Glucose Metabolism

The association between SHS and glucose metabolism was analyzed. The levels of FPG and insulin were used as indexes for evaluating glucose metabolism.

**Figure 2:**
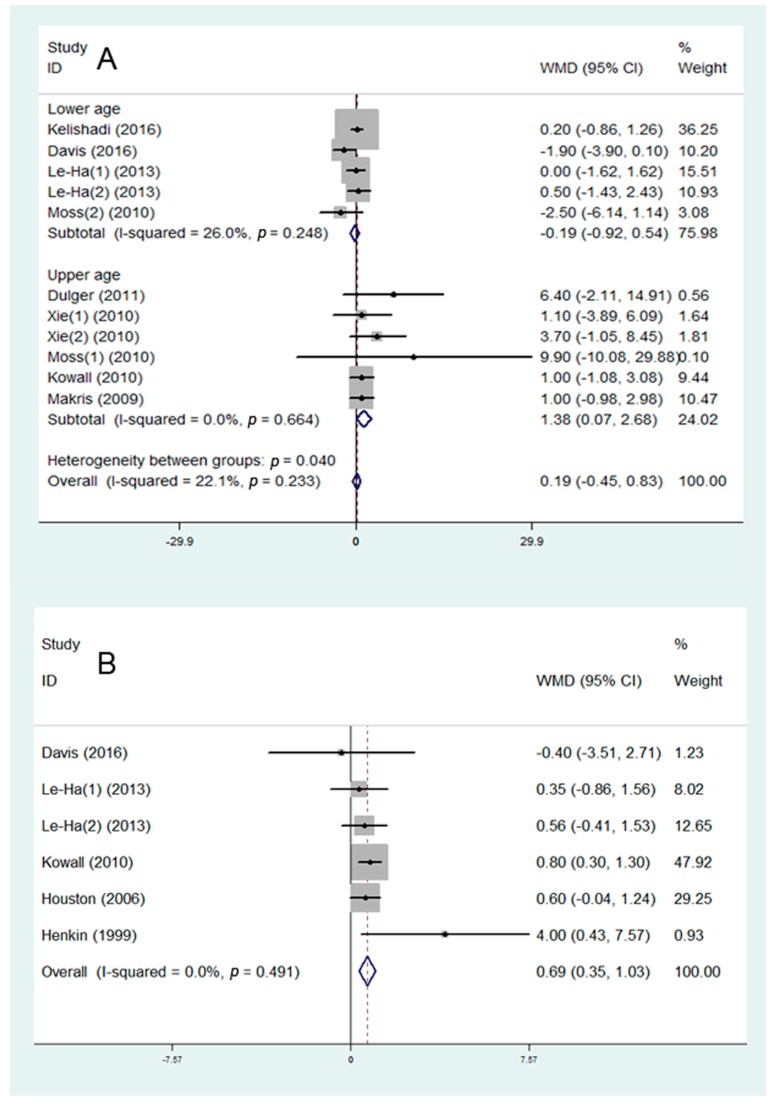
Meta-analysis of secondhand smoke (SHS) and risk of glucose metabolism disorder: (**A**) association between SHS and fasting plasma glucose (FPG); (**B**) association between SHS and insulin. WMD—weighted mean difference.

Eight studies were included in the assessment of the association between SHS and FPG. The studies were divided into two subgroups: lower age group (7–18 years) and upper age group (27–74 years). It was found that SHS may be positively associated with the FPG in the upper age group (WMD = 1.38 mg/dL, 95% CI: 0.07 to 2.68 mg/dL, I^2^ = 0.0%, Figure 2A), but showed no significant influence on young people. Five studies were included in the assessment of the association between SHS and insulin. It was shown that SHS may be positively associated with the level of insulin (WMD = 0.69 mU/L, 95% CI: 0.35 to 1.03 mg/dL, I^2^ = 0.0%, Figure 2B).

### 3.4. SHS and Disorder of Lipid Metabolism

The association between SHS and lipid metabolism was analyzed. The levels of total cholesterol, triglycerides, LDL-C, and HDL-C were used as indexes for evaluating lipid metabolism. All studies were divided into two subgroups: lower age group (10–18 years) and upper age group (total cholesterol: 30–60 years; triglycerides: 18–60 years; LDL-C: 26–60 years; HDL-C: 30–60 years).

**Figure 3:**
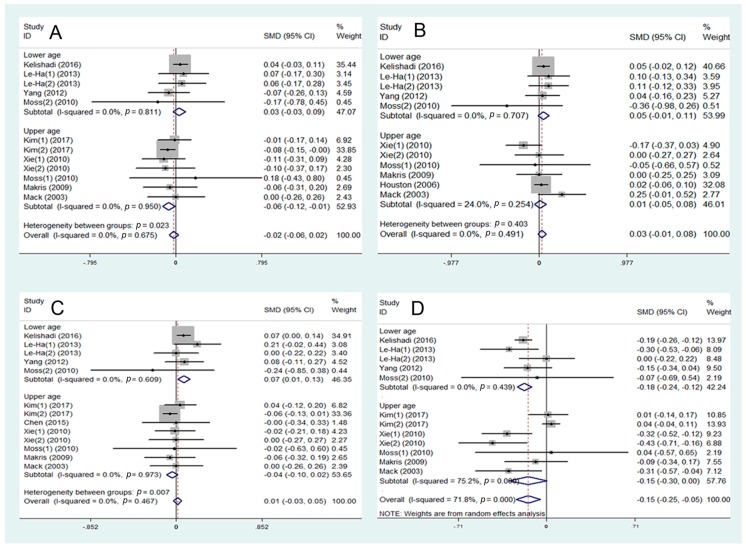
Meta-analysis of secondhand smoke (SHS) and risk of lipid metabolism disorder: (**A**) association between SHS and total cholesterol; (**B**) association between SHS and triglycerides; (**C**) association between SHS and LDL-C; (**D**) association between SHS and HDL-C. WMD—weighted mean difference; SMD—standard mean difference.

Eight studies were included in the assessment of the association between SHS and total cholesterol. A negative association was found between SHS and total cholesterol in the upper age group (SMD = –0.06, 95% CI: –0.12 to –0.01, I^2^ = 0.0%, Figure 3A). Nine studies were included in the assessment of the level of LDL-C. SHS may be positively associated with LDL-C level in the lower age group (SMD = 0.07, 95% CI: 0.01 to 0.13, I^2^ = 0.0%, Figure 3C), but has no effect in the upper age group. Eight studies were included in the assessment of HDL-C. A negative-related effect of SHS on HDL-C may exist in the lower age group (SMD = –0.18, 95% CI: –0.24 to –0.12, I^2^ = 75.2%, Figure 3D). No significant association was found between SHS and triglycerides (Figure 3B).

### 3.5. SHS and Risk of Abdominal Obesity

The association between SHS and the risk of abdominal obesity was analyzed. BMI and WC were used as indexes for evaluating lipid metabolism.

**Figure 4:**
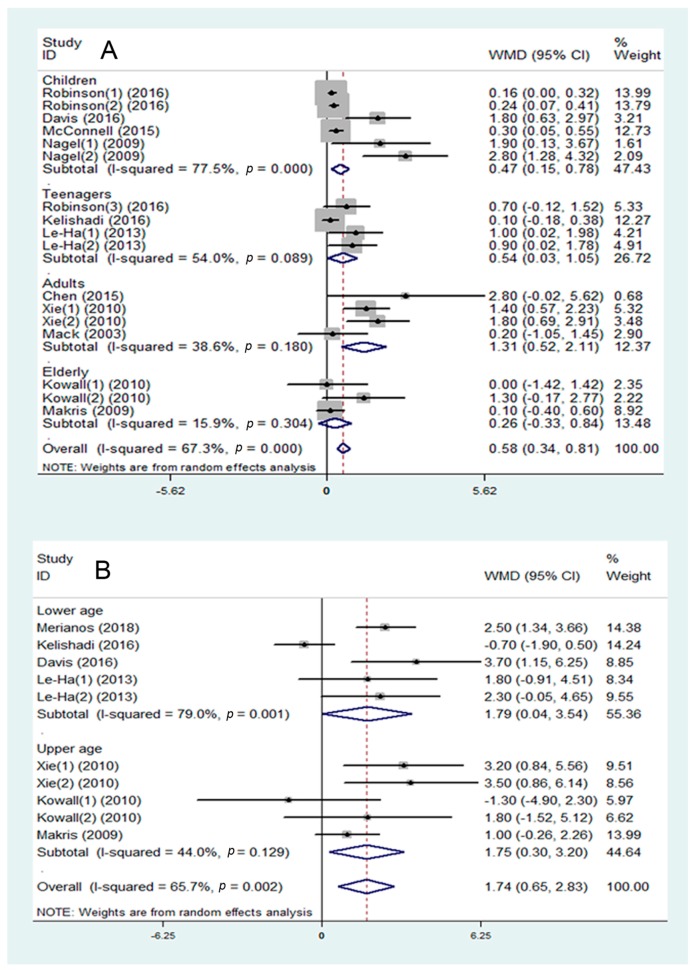
Meta-analysis of secondhand smoke (SHS) and risk of abdominal obesity: (**A**) association between SHS and body mass index (BMI); (**B**) association between SHS and waist circumference (WC). WMD—weighted mean difference; SMD—standard mean difference.

Eleven studies were included in the assessment of BMI, and they showed that SHS may be associated with a higher BMI (WMD = 0.58 kg/m^2^, 95% CI: 0.34 to 0.81 kg/m^2^, I^2^ = 67.3%, Figure 4A). In the subgroup analysis, data were divided into four groups: children (1–10 years), teenagers (10–18 years), adults (26–50 years), and elderly (50–74 years). SHS showed different degrees of impact on the four groups. In the adults group, there was a stronger association between SHS and BMI (WMD = 1.31 kg/m^2^, 95% CI: 0.52 to 2.11 kg/m^2^, I^2^ = 38.6%, Figure 4A) than in the children group (WMD = 0.47 kg/m^2^, 95% CI: 0.15 to 0.78 kg/m^2^, I^2^ = 77.5%, *p* < 0.05, Figure 4A), and it showed stronger trends than in the teenagers group (WMD = 0.54 kg/m^2^, 95% CI: 0.03 to 1.05 kg/m^2^, I^2^ = 54.0%, 0.05 < *p* < 0.10, Figure 4A). No significant effects were shown in the elderly group.

Seven studies were included in the assessment of the association between SHS and WC. It was found that SHS may be associated with a higher WC (WMD = 1.74 cm, 95% CI: 0.65 to 2.83 cm, I^2^ = 65.7%, Figure 4B). All studies were divided into two subgroups: lower age group (7–19 years) and upper age group (30–74 years). In the subgroup analysis, SHS was positively associated with WC in both the lower age group (WMD = 1.79 cm, 95% CI: 0.04 to 3.54 cm, I^2^ = 79.0%, Figure 4B) and the upper age group (WMD = 1.75 cm, 95% CI: 0.30 to 3.20 cm, I^2^ = 44.0%, Figure 4B).

## 4. Discussion

Metabolic syndrome is a combination of metabolic abnormalities. Kaur et al. reported that the global prevalence of metabolic syndrome ranges from 10% to as much as 84%, depending on the ethnicity, age, gender, and race of individuals in the population [37]. Patients with metabolic syndrome are at high risk for developing diabetes, cardiovascular disease, and a plethora of cancers, and they have an increased rate of mortality [38,39,40]. The main features of the metabolic syndrome include glucose and lipid metabolism disorders, insulin resistance, and abdominal obesity. In terms of glucose metabolism, levels of FPG and insulin are often used as indicators. Our study found that SHS may be positively associated with FPG in the upper age group. The levels of insulin were also positively associated with SHS exposure. Thus, it can be speculated that adults may be more sensitive than minors in terms of the effects of SHS on glucose metabolic disorder. In terms of lipid metabolism, lipid (total cholesterol, triglycerides, HDL-C, LDL-C) levels are usually analyzed. Our study found that SHS was positively associated with the level of LDL-C and negatively associated with the level of HDL-C in the lower age group. Therefore, it can be speculated that the risk of dyslipidemia caused by SHS is higher among younger people than among the older ones. According to our meta-analysis, SHS has a positive impact on BMI. In different age groups, SHS showed the highest degree of impact on BMI values of people aged 26 to 50 years. However, BMI cannot represent abdominal obesity completely, as it gives no indication of distribution of body fat. WC is a highly sensitive and specific measure of abdominal fat accumulation and thus it is valuable for identifying individuals with abdominal obesity who are at risk of developing metabolic complications [41]. Our study also found that SHS was positively associated with WC. Therefore, it is suggested that exposure to SHS is associated with an increased risk of developing abdominal obesity. In summary, we suggest that SHS may be closely related to the development of metabolic syndrome.

Next, we will try to further explain the age difference that exists in our results. In our study, the level of FPG was increased in older people but showed no effect in younger people. The balance between insulin secretion from β-cells and peripheral insulin sensitivity is what maintains normal glucose homeostasis [42]. It is reported that the ability to regulate glucose is progressively lost with age [43]. The actions of insulin and β-cell function also decrease with age [44,45]. Therefore, SHS may more easily destroy glucose homeostasis in older people and aggravate the age-related disorder of glucose metabolism. In younger people, the body’s glucose metabolism follows a normal process—insulin secretion from β-cells is tightly coupled to the availability of glucose, allowing glucose to be maintained within a stable and normal range [42]. When young people are exposed to SHS, the influence may be dynamically regulated by homeostasis mechanisms of their bodies.

In our meta-analysis, the positive association between SHS and levels of LDL-C, and the negative association between SHS and HDL-C were more obvious in younger people than in older people. According to Murakata et al.’s study, the serum concentration of LDL-C is positively correlated with age, and HDL-C is negatively correlated with age [46]. These changes that occur with increasing age are consistent with the trends of blood lipids caused by SHS in our hypothesis. Therefore, it can be speculated that the effects of SHS on blood lipids in older people might be covered by age-related effects. In addition, children and teenagers are at a critical stage of growth and development, and their blood lipids are susceptible to living environmental factors, such as unhealthy diets, smoking, pesticides, and metals [19,47,48]. This may explain why SHS is more likely to lead to significant dyslipidemia in younger people than in older people.

Our study focused on a very timely public health topic—metabolic disorder. It is the first study to use a meta-analysis to review the potential association of SHS with metabolic syndrome and to explain the possible age differences. The included studies are up-to-date and of sufficient quality. Abundant indicators to evaluate metabolic syndrome were used in our study. Our study still needs some improvements—more databases should be included in our study and more subgroups could be analyzed.

## 5. Conclusions

In conclusion, the association of metabolic syndrome with SHS varies with age. When exposed to SHS, older people may be more susceptible to glucose metabolic disorder, but younger people may be more susceptible to lipid metabolic disorder.

## Figures and Tables

**Table 1:** Characteristics of included studies.

Study	Study Period	Study Design	SHS Assessment	Mean Age (Year)	Sample Size	Variables
Merianos et al. (2018) [19]	1999–2012	CS	Non-smoker, serum cotinine (0.05–2.99 ng/mL)	12–19	11,550	WC
Kim et al. (2017) [20]	2008–2011	CS	Self-report passive smoking	45	7376	TC, LDL-C, HDL-C
Robinson et al. (2016) [21]	2003–2008	C	Questionnaire: non-smoker, passive exposure at home/work	1/4/14	3174	BMI
Kelishadi et al. (2016) [22]	2009–2010	CS	Self-report: living with smokers	10–18	5625	BMI, WC, FPG, TC, TG, LDL-C, HDL-C
Davis et al. (2016) [23]	2003–2006	CS	Non-smoker, plasma cotinine ≥ 0.05 ng/mL	7–11	222	BMI, WC, INS, FPG
McConnell et al. (2015) [24]	NC	C	Questionnaire: living with smokers, quantity of smokers in the household	10	3318	BMI
Chen et al. (2015) [25]	2004–2010	C	Questionnaire: non-smoker, living together indoors with smokers, the number of exposed years	26–48	415	BMI, LDL-C
Le-Ha et al. (2013) [26]	1989–2006	C	Questionnaire: quantity of cigarettes smoked daily in the household	17	1754	BMI, WC, INS, FPG, TC, TG, LDL-C, HDL-C
Yang et al. (2012) [27]	NC	CS	Non-smoker, plasma cotinine ≥ 0.90 ng/mL	16	624	TC, TG, LDL-C, HDL-C
Dulger et al. (2011) [28]	NC	CS	Non-smoker, living with smokers for at least 5–6 h during the day	27–33	40	FPG
Xie et al. (2010) [29]	NC	CS	Questionnaire: number of days/one week in a room with smokers	38	389	BMI, WC, FPG, TC, TG, LDL-C, HDL-C
Moss et al. (2010) [30]	NC	CS	Self-report passive smoking	30–50/10–12	41	FPG, TC, TG, LDL-C, HDL-C
Kowall et al. (2010) [31]	1999–2001	C	Questionnaire: stay with smokers at home/work, quantity of other smokers	55–74	1223	BMI, WC, INS, FPG
Nagel et al. (2009) [32]	1999–2008	CS	Questionnaire: living with smokers, quantity of cigarettes smoked daily in the household	10	450	BMI
Makris et al. (2009) [33]	NC	CS	Non-smoker, at least 1 h daily domestic and/or workplace smoke exposure	50–60	254	BMI, WC, FPG, TC, TG, LDL-C, HDL-C
Houston et al. (2006) [34]	NC	C	Self-report and serum cotinine (1–15 ng/mL)	18–30	4572	INS, TG
Mack et al. (2003) [35]	NC	CS	Questionnaire: number of smokers and hours per day staying with smokers	40	227	BMI, TC, TG, LDL-C, HDL-C
Henkin et al. (1999) [36]	NC	CS	Structured interview: living with smokers	40–69	1481	INS

SHS—Secondhand smoke; NC—Not Clear; CS—Cross-sectional Study; C—Cohort Study; BMI—Body Mass Index; WC—Waist Circumference; INS—Insulin; FPG—Fasting Plasma Glucose; TC—Total Cholesterol; TG—Triglycerides; LDL-C—Low Density Lipoprotein Cholesterol; HDL-C—High Density Lipoprotein Cholesterol.

**Table 2:** Quality assessment of included studies.

**Cross-Sectional Study**	**Year**	**Research Elements**				**Quality Control**				**Data Integrity**			**Scores**	Quality *
		**1**	**2**	**3**	**4**	**5**	**6**	**7**	**8**	**9**	**10**	**11**		
Merianos	2018	★	★	★	★	-	★	★	★	-	★	-	8	High
Kim	2017	★	★	★	★	★	★	★	★	-	-	-	8	High
Kelishadi	2016	★	-	★	-	-	★	-	★	-	★	-	5	Moderate
Davis	2016	★	★	★	-	★	★	-	★	-	-	★	7	Moderate
Yang	2012	★	★	-	-	★	★	★	★	-	-	-	6	Moderate
Dulger	2011	★	★	-	-	★	★	★	★	-	-	-	6	Moderate
Xie	2010	★	★	★	-	★	★	★	★	★	★	-	9	High
Moss	2010	★	★	-	-	★	★	★	★	★	★	-	8	High
Nagel	2009	★	★	★	-	★	-	★	★	-	★	★	8	High
Makris	2009	★	★	-	★	★	★	★	★	-	★	★	9	High
Mack	2003	★	-	-	-	★	★	★	★	-	★	-	6	Moderate
Henkin	1999	★	★	-	-	★	★	★	★	-	★	★	8	High
**Cohort Study**	**Year**	**Selection**				**Comparability**			**Result Evaluation**				**Scores**	Quality *
		**I**	**II**	**III**	**IV**	**V**			**VI**	**VII**	**VIII**			
Robinson	2016	★	★	-	★	★★			★	★	★		8	High
McConnell	2015	-	★	-	★	★★			★	★	★		7	High
Chen	2015	★	★	-	★	★★			★	★	★		8	High
Le-Ha	2013	★	★	★	★	★★			★	★	★		9	High
Kowall	2010	★	★	-	★	★★			-	★	★		7	High
Houston	2006	★	★	★	★	★★			-	★	★		8	High

(1) Define the source of information; (2) list inclusion and exclusion criteria for exposed and unexposed subjects; (3) list the time period for identifying patients; (4) indicate whether subjects were consecutive if not population based; (5) indicate whether subjective components of evaluators were masked by other aspects of participants; (6) describe quality assurance purposes; (7) explain any patient exclusions; (8) describe how confounding was assessed or controlled; (9) explain how missing data were handled; (10) summarize patient response rates and the completeness of the data collection; (11) provide follow-up information and percentage of incomplete data. (I) Representativeness of the exposed cohort; (II) selection of non-exposed cohort; (III) ascertainment of exposure; (IV) demonstration that the outcome of interest was not present at the start of the study; (V) comparability of cohorts on the basis of the design or analysis; (VI) assessment of outcome; (VII) was follow-up long enough for outcomes to occur; (VIII) adequacy of follow-up of cohorts. ★—Got one score in this item; *—The quality of cross-sectional studies was evaluated by the Agency for Healthcare Research and Quality (AHRQ), and the quality of cohort studies was evaluated by the Newcastle-Ottawa Scale (NOS) assessment.
